# The Effect of Tellurite on Highly Resistant Freshwater Aerobic Anoxygenic Phototrophs and Their Strategies for Reduction

**DOI:** 10.3390/microorganisms3040826

**Published:** 2015-11-06

**Authors:** Chris Maltman, Vladimir Yurkov

**Affiliations:** Department of Microbiology, University of Manitoba, Winnipeg, MB R3T 2N2, Canada; E-Mail: ummaltma@myumanitoba.ca

**Keywords:** tellurite, aerobic anoxygenic phototrophs, metalloid oxyanions, metalloid transformation, tellurite reduction

## Abstract

Six fresh water aerobic anoxygenic phototrophs (*Erythromicrobium ezovicum*, strain E1; *Erythromicrobium hydrolyticum*, E4(1); *Erythromicrobium ramosum*, E5; *Erythromonas ursincola*, KR99; *Sandaracinobacter sibiricus*, RB 16-17; and *Roseococcus thiosulfatophilus*, RB3) possessing high level resistance to TeO_3_^2−^ and the ability to reduce it to elemental Te were studied to understand their interaction with this highly toxic oxyanion. Tested organic carbon sources, pH, and level of aeration all had an impact on reduction. Physiological and metabolic responses of cells to tellurite varied among strains. In its presence, *versus* absence, cellular biomass either increased (KR99, 66.6% and E5, 21.2%) or decreased (RB3, 66.1%, E1, 57.8%, RB 16-17, 41.5%, and E4(1), 21.3%). The increase suggests a possible benefit from tellurite. Cellular ATP production was similarly affected, resulting in an increase (KR99, 15.2% and E5, 38.9%) or decrease (E4(1), 31.9%; RB 16-17, 48.8%; RB3, 55.9%; E1, 35.9%). Two distinct strategies to tellurite reduction were identified. The first, found in E4(1), requires *de novo* protein preparations as well as an undisturbed whole cell. The second strategy, in which reduction depended on a membrane associated constitutive reductase, was used by the remaining strains.

## 1. Introduction

Tellurium (Te) is a group 16 metalloid element related to sulphur and oxygen. It possesses stable oxidation states of +VI (tellurate), +IV (tellurite), 0 (elemental Te), and −II (telluride). The majority is found in the hydrosphere as tellurate and in the lithosphere as tellurides of gold and silver [1]. The most harmful forms of Te to microorganisms are the oxyanions, especially tellurite, with concentrations as low as 1 µg/mL being highly toxic [2]. However, the ability to reduce it to the elemental form allows certain bacterial species to resist concentrations up to 4000 µg/mL [3]. The means by which this compound exerts its toxicity is still debated, however, the strong oxidative properties [4], confirmed by an E° of 0.827 V for the TeO_3_^−2^/Te redox couple [5], are likely among the reasons.

Tellurite exposure can cause the formation of intracellular radical oxygen species (ROS) [6], leading to cellular damage. Catalases, the key enzymatic defence against ROS, play a role in mitigating the detrimental effects of the toxin. In *Staphylococcus epidermidis*, this family of enzymes is also capable of using TeO_3_^2−^ as a substrate, reducing it to Te [7], therefore minimizing the negative impact on cells. In *E. coli*, reduction of tellurite can occur through the actions of nitrate reductases, however, this is a non-specific reaction [8,9]. Other enzymes, such as the thiol:disulfide oxidoreductase of *Rhodobacter capsulatus* [10], GutS from *E. coli* [11], among others [12,13,14,15] have been implicated in tellurite resistance and/or reduction. Although they are associated with low to moderate levels of resistance, it is not their primary specific function. In the case of *R. capsulatus*, multiple approaches to dealing with tellurite have been observed. One involves maintaining redox poise during photosynthetic growth through the reduction of tellurite [16], while another is based on reduced uptake of the tellurite oxyanion. With the latter, acetate permease is responsible for TeO_3_^2−^ influx [17] and competition between it and acetate for entry into the cell results in higher resistance. Even at low concentrations (60 ng/mL), acetate impacts tellurite entry [18], limiting toxicity. A related approach has been identified in *E. coli*. Mutation to a phosphate transport system provided enhanced resistance [19], which allowed tellurite ingress. Lastly, certain microorganisms can somewhat neutralize Te oxyanions by production of volatile organic telluride compounds, such as dimethyltelluride [20], however, such approach to detoxification delivers negligible removal.

While the aforementioned physiological reactions are utilized for TeO_3_^2−^ resistance and/or reduction, none involves a specific tellurite reductase. Our understanding of how bacteria carry this out is limited. Unlike for selenate resistance/reduction, where specific reductases have been identified, such in cells of *Thauera selenatis* [21], only a single example of a tellurite specific reductase has been isolated to date from the *Bacillus* sp. STG-83 [22]. Although not proven, this bacterium might be capable of dissimilatory anaerobic reduction, therefore, the enzyme is likely respiratory in nature. Investigation into the strategies for tellurite reduction has just begun to expand. Recently, several bacterial species have been isolated that are highly resistant to tellurite (up to 2700 µg/mL) [23,24]. Among bacteria possessing very high level resistance are aerobic anoxygenic phototrophs (AAP) isolated from extreme environments [25]. This group of bacteria seems to have evolved an inherent ability to deal with this oxyanion. Therefore, we set forth to investigate the physiological and metabolic effects of TeO_3_^2−^ on cells, factors affecting reduction, and differences in expression of a reducing system by AAP inhabiting extreme environments. Species chosen were *Erythromicrobium ezovicum* (strain E1), *Erythromicrobium hydrolyticum* (E4(1)), *Erythromicrobium ramosum* (E5), *Erythromonas ursincola* (KR99), *Sandaracinobacter sibiricus* (RB 16-17), and *Roseococcus thiosulfatophilus* (RB3). All are freshwater bacteria from cyanobacterial mats developed around thermal springs in the Baikal Lake region in Russia [26,27,28,29,30]. They reduce very high levels of tellurite to elemental Te under aerobic conditions [2].

## 2. Experimental Section

### 2.1. Strains and Growth Conditions

Bacteria chosen for study include *Erythromicrobium ezovicum* (strain E1), *Erythromicrobium hydrolyticum* (E4(1)), *Erythromicrobium ramosum* (E5), *Erythromonas ursincola* (KR99), *Sandaracinobacter sibiricus* (RB 16-17), and *Roseococcus thiosulfatophilus* (RB3) [26,27,28,29,30]. They were grown aerobically in the dark at their optimal temperature (28 °C) on an incubator shaker (200 rpm) in liquid rich organic (RO) or liquid minimal salts (MS) media [30,31] containing either glutamate, pyruvate, and malate or glutamate, pyruvate, and yeast extract each at 1.5 g/L, at pH 9.0 unless otherwise stated. All results are an average of three replicates.

### 2.2. Physiological and Biochemical Tests

Metalloid resistance, utilization of organic substrates, variation in pH, level of aeration, and protein and ATP production were all examined in the presence of K_2_TeO_3_. Resistance was confirmed in RO liquid medium with varying concentrations of K_2_TeO_3_ (100, 250, 500, 750, 1000, and 1500 µg/mL). Growth was monitored spectrophotometrically at A_950_, an established method for estimating growth and reduction in the presence of tellurite, over 96 h [2]. All growth for subsequent experiments was monitored at A_950_ with 500 µg/mL (strains E1, E4(1), E5, and KR99) or 100 µg/mL (RB3 and RB 16-17) K_2_TeO_3_ in liquid culture over 96 h, unless otherwise described. The effect of carbon sources on growth and reduction was investigated by transfer of actively growing cells to MS liquid medium, pH 7.8, with K_2_TeO_3_ containing one of: acetate, butyrate, citrate, ethanol, fructose, glucose, glutamate, L-glutamine, lactate, malate, pyruvate, or succinate at either 1.5 or 3.0 g/L. The solubility of K_2_TeO_3_, and therefore the availability in solution, changes with pH [32] that is why the effect of pH on resistance and reduction was tested. As the addition of K_2_TeO_3_ to the growth medium caused the formation of precipitates under acidic conditions and strains could not grow beyond pH 9.5, only pH range 7.0 to 9.0 was considered. Liquid medium was adjusted with 0.5 N NaOH to the desired pH. The role of oxygenation was analyzed in an incubator shaker set to 100 (low), 200 (typical) or 300 (high) rpm. Once optimal conditions were established, strains were grown at those parameters with 500 or 1000 µg/mL (strains E1, E4(1), E5, and KR99) or 100 or 500 µg/mL (RB3 and RB 16-17) K_2_TeO_3_. To observe the effect of tellurite on cellular protein and ATP levels, measurements were taken in its presence and absence over 48 h. Protein was assayed by the Bradford method [33] and ATP was monitored with an ATP Bioluminescence Kit from Sigma-Aldrich, following extraction from samples with perchloric acid [34].

### 2.3. Tellurite Reductase Expression, Activity, and Localization

Tellurite reductase expression experiments were carried out as recently published [35], with one modification: 100 µg/mL chloramphenicol was used for strain RB 16-17 instead of tetracycline. Detection of TeO_3_^2−^ reduction in cell extracts and localization of reductase activity was performed as described [35]. Rate of reduction (1 unit equal to 1 µg tellurite reduced/µg protein/h) was calculated for each cellular fraction. For isolation of membranes, cells were broken by French Press and centrifuged at 20,000 rpm for 1 h to remove debris. The supernatant was collected and ultracentrifuged at 60,000 rpm for 12 h. The membrane pellet was then washed with 10 mM Tris HCl, pH 8.0. Membranes were resuspended in their respective growth media containing K_2_TeO_3_.

## 3. Results

### 3.1. Growth with Tellurite

Growth of AAP in the presence of different K_2_TeO_3_ concentrations confirmed these bacteria possess a high level resistance. Strains appear to be similar, resisting and reducing up to 1500 µg/mL, however, optimal growth and reduction occurred at 500 µg/mL K_2_TeO_3_ for E1, E4(1), E5, and KR99, while 100 µg/mL K_2_TeO_3_ was best for RB3 and RB 16-17. With respect to media composition, we observed that it does have an effect, as has been previously reported [2]. Although many different carbon sources and combinations were tested, only three media compositions gave optimal growth and reduction. Strains E4(1), RB3, and RB 16-17 performed best in complex RO medium, while E5 and KR99 preferred defined MS medium containing a combination of glutamate, malate, and pyruvate. E1 showed the best results in MS medium with glutamate and pyruvate, however, some yeast extract was still required, suggesting a need for the undefined component of this complex organic substrate. To determine if the specific combination, but not the increased organics, was responsible for increased growth and reduction with tellurite, each individual source was tested at 3.0 g/L. Single organic carbon sources at increased concentrations were not as good as the combination. All tested strains grew and reduced K_2_TeO_3_ optimally at pH 9.0 and aeration was achieved at 200 rpm (Table 1). However, reduction still occurred at 100 and 300 rpm, albeit at much reduced levels.

**Table 1:** Effect of pH and aeration on growth and reduction of K_2_TeO_3_ estimated at A_950_ over 96 h and represented as percent of maximal growth.

Strain	pH			Aeration (rpm)		
	7.0	8.0	9.0	100	200	300
E1 ^1^	78.4 ± 3.8	82.5 ± 4.8	100 ± 1.8	61.8 ± 5.9	100 ± 5.4	64.5 ± 4.9
E4(1) ^1^	68.1 ± 5.1	77.6 ± 2.9	100 ± 0.7	54.2 ± 4.6	100 ± 2.2	67.4 ± 3.5
E5 ^1^	62.8 ± 4.5	74.9 ± 2.4	100 ± 4.7	81.7 ± 3.7	100 ± 2.7	72.1 ± 2.9
KR99 ^1^	77.7 ± 1.9	97.6 ± 1.1	100 ± 1.5	47.6 ± 2.2	100 ± 3.2	53.9 ± 4.4
RB 16-17 ^2^	70.8 ± 3.3	71.4 ± 4.6	100 ± 4.3	55.9 ± 2.9	100 ± 3.7	66.8 ± 3.3
RB3 ^2^	78.2 ± 3.6	76.6 ± 5.5	100 ± 3.2	59.7 ± 3.1	100 ± 4.3	62.1 ± 3.6

^1^ 500 µg/mL tellurite added; ^2^ 100 µg/mL tellurite added.

### 3.2. Effect of Tellurite on Protein and ATP Production

**Figure 1:**
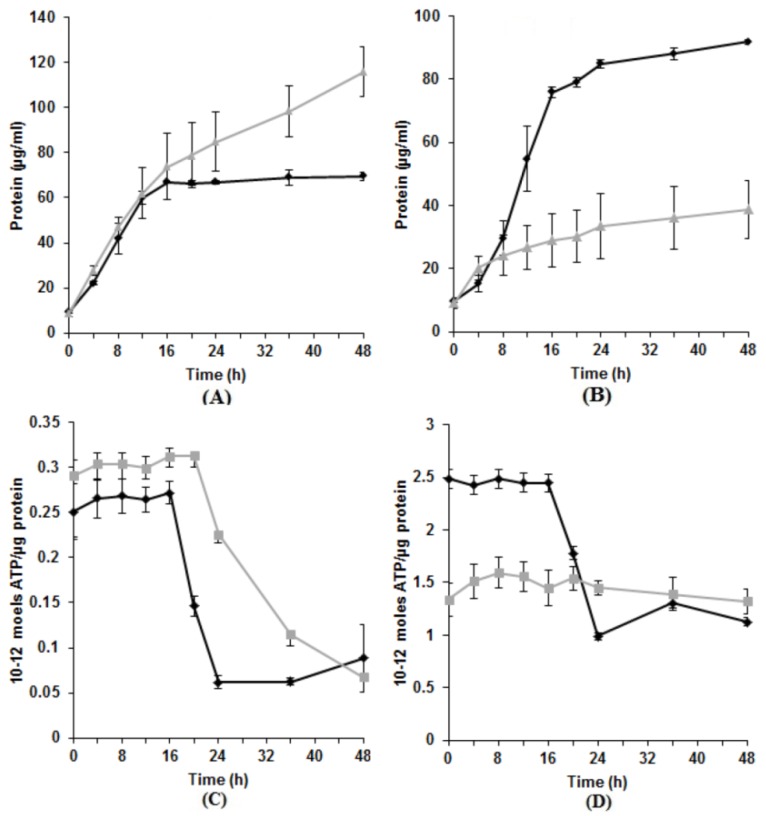
Protein and ATP production in the presence *versus* absence of K_2_TeO_3_. (**A**) Strain KR99. Similar results for E5; (**B**) Strain E1. Similar results for E4(1), RB3, and RB 16-17. ♦—No K_2_TeO_3_; ▲—500 µg/mL K_2_TeO_3_; (**C**) Strain KR99. Similar results for E5; (**D**) Strain E1. Similar results for E4(1), RB3, and RB 16-17. ♦—No K_2_TeO_3_; ▀—500 µg/mL K_2_TeO_3_. Error bars represent one standard deviation.

While it has been shown that proteins, specifically those containing reduced thiol groups, can be damaged by TeO_3_^2−^ [36], its direct effect on highly resistant bacteria is unclear. With the majority of known impacts of tellurite on cells being negative [36], except for the select few species that can anaerobically respire on TeO_3_^2−^ [37,38], we expected there would be reduced growth and, therefore, decreased protein in its presence. Indeed, strains E1, E4(1), RB3, and RB 16-17 showed a drop in protein production as expected (57.8%, 21.3%, 66.1%, and 41.5%, respectively) (Figure 1B). However, KR99 and E5 had an increase in protein levels (66.6% and 21.2%, respectively) (Figure 1A).

Exposure to tellurite in aerobically grown *E. coli* causes a loss of the transmembrane proton gradient leading to depletion of intracellular ATP [39]. Therefore, we predicted that ATP levels in our experiments might be decreased. As expected, this was observed for E1, E4(1), RB3, and RB 16-17 (35.9%, 31.9%, 55.9%, and 48.8% decrease per unit protein, respectively) (Figure 1D). However, unexpectedly, E5 and KR99 cells produced higher levels of ATP in the presence of tellurite (38.9% and 15.2% increase, respectively) (Figure 1C). For these two strains, an increase was measured in both protein and ATP, and for E1, E4(1), RB3, and RB 16-17 a decrease in each case.

### 3.3. Characteristics of Tellurite Reductase Activity

Generally, if a bacterium must induce expression of a protein to cope with the presence of a harmful substance, a lag phase will be observed during a primary exposure, while the specific product/enzyme is being prepared [40]. However, during subsequent exposure, a lag phase is usually unnecessary, since everything required for synthesis is ready. Such phenomenon has been observed in experiments with tellurite [35] as well as some other metal(loid) oxyanions, for example U(VI) [41]. To determine if tellurite reduction involves *de novo* production of a specific enzyme, growth physiology during primary and secondary exposure was compared. If growth parameters in both cases were similar, it is likely that the reducing enzyme was constitutively present. However, a lag phase detected during primary exposure, but absent in secondary, would imply the need for initiation of transcription. Strains E1, E5, KR99, RB3, and RB 16-17 all possessed similar growth rates during both primary and secondary exposure (Figure 3A), indicating a constitutive reductase. The remaining strain E4(1) was the exception. Growth was significantly hindered during secondary exposure (Figure 3B). As a result, it could not be determined if there was a lag phase during primary exposure.

**Figure 2:**
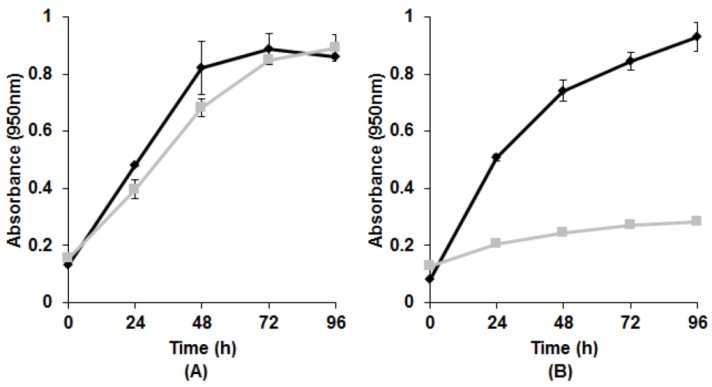
Growth and reduction during primary *vs.* secondary exposure to K_2_TeO_3_. (**A**) Strain KR99. Similar results for E1, E5, RB3, and RB 16-17; (**B**) Strain E4(1). ♦—Primary exposure; ▀—Secondary exposure. Error bars represent one standard deviation.

**Figure 3:**
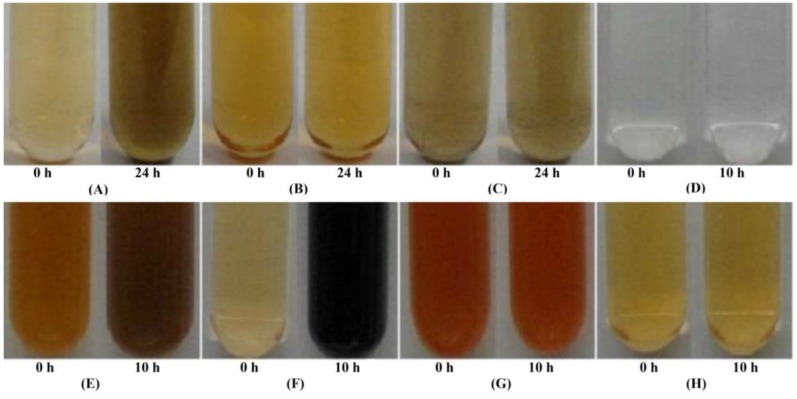
Reductase activity in cellular fractions. (**A**) Cell lysate of strain E1 grown without prior exposure to K_2_TeO_3_. Similar results were found for KR99, E5, RB3, and RB 16-17; (**B**) Lysate of strain E4(1) grown without prior exposure to K_2_TeO_3_; (**C**) Lysate of E4(1) cells grown with prior exposure to K_2_TeO_3_. Initial darkening at 0 h is due to the trace presence of previously reduced K_2_TeO_3_ from prior exposure; (**D**) Periplasmic fraction of KR99 without K_2_TeO_3_ exposure. No reductase activity observed. Similar results for E5, E4(1), E1, RB3 and RB 16-17; (**E**) Spheroplast fraction of E1 without prior K_2_TeO_3_ exposure containing reductase activity. Similar results for E5, KR99, RB3, and RB 16-17; (**F**) Spheroplast lysate of KR99 without prior K_2_TeO_3_ exposure containing reductase activity. Similar results for E5, E1, RB3, and RB 16-17; (**G**) E4(1) spheroplast fraction. No reductase activity observed with or without prior K_2_TeO_3_ exposure; (**H**) E4(1) spheroplast lysate. No reductase activity observed with or without prior K_2_TeO_3_ exposure.

Upon halting protein synthesis with tetracycline or chloramphenicol, we found strains E1, E5, KR99, RB3, and RB 16-17 were still capable of reducing tellurite, supporting a constitutive system. In cells of E4(1) reduction was inhibited, indicating *de novo* preparations are required. To further support the idea that the enzyme(s) responsible for reduction are constitutive, reduction in the cell lysates was analyzed (Figure 3). Lysates of E1, E5, KR99, RB3, and RB 16-17 cells were capable of reducing K_2_TeO_3_ without prior exposure (Figure 3A), whereas those of E4(1) could not (Figure 3B), even following exposure (Figure 3C).

### 3.4. Localization of Reductase Activity

**Figure 4:**
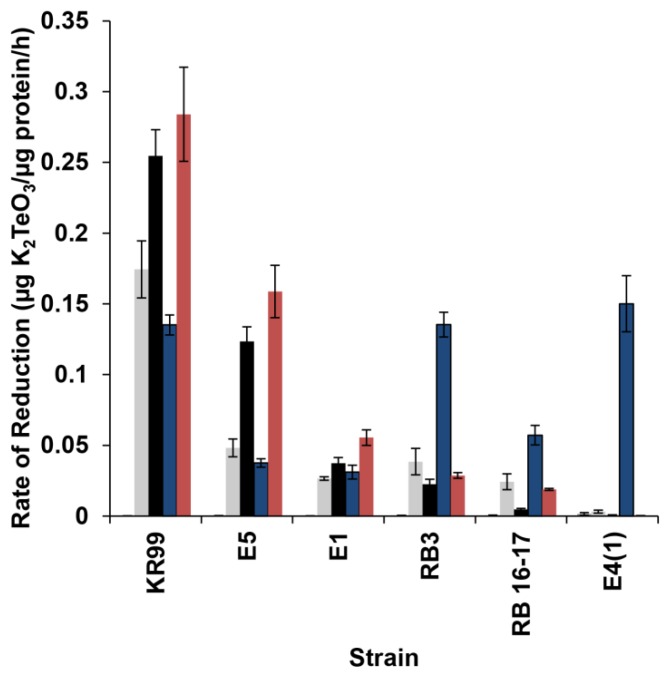
Rate of K_2_TeO_3_ reduction in cellular fractions. ▀ Periplasm, ▀ Spheroplast, ▀ Spheroplast Lysate, ▀ Whole Cells, ▀ Membranes. Error bars represent one standard deviation.

While all strains, with the exception of E4(1), possessed a constitutive tellurite reductase, its location was unknown. Therefore, strains were fractionated and each fraction monitored for reduction. No reductase activity was observed in the periplasm of any strain (Figure 3D), however, E1, E5, KR99, RB3, and RB 16-17 all possessed activity in the spheroplast and spheroplast lysate (Figure 3E,F). In the case of E4(1), no activity was observed in any fraction with or without prior exposure to TeO_3_^2−^ (Figure 3G,H), strictly confirming a possibility of reduction in intact cells only. Upon separation of the membranes from the cytoplasmic contents, activity was detected for E1, E5, KR99, RB3, and RB 16-17. The rate of reduction was calculated for each fraction (Figure 4). Strain KR99 possessed the highest rate of 0.284 units in the membranes. The strain with the second highest rate was E5 (0.159 units in the membranes), followed by E1 (0.056 units in the membranes), RB3 (0.038 units in spheroplast), and RB 16-17 (0.024 units in spheroplast).

## 4. Discussion

Our understanding of AAPs in general [42,43] and their interaction with tellurite in particular is still poor with many questions remaining. Obviously, the composition of the growth medium influences the level of resistance. Previous work has shown that in complex medium resistance to tellurite can be increased [2]. In this study we found a similar trend, with E1, E4(1), RB3, and RB 16-17 favoring RO medium. However, E5 and KR99 preferred a defined medium containing glutamate, pyruvate, and malate. We do not know why such a difference was seen. Perhaps these carbon sources are involved with, or can be directly utilized by, the TCA cycle [44], providing an advantage in energy generation under the stress of K_2_TeO_3_ pressure. Therefore, with conditions conducive to easier growth, the negative impact of tellurite can be overcome. It is also likely that these compounds are acting similar to how acetate does in *R. capsulatus*, competing with tellurite for cellular entry, thereby increasing resistance [17,18].

The impact of TeO_3_^2−^ on cellular proteins generally, as expected, resulted in decrease. However, there were also some unexpected turns. Strains E5 and KR99 surprisingly produced more protein in the presence of tellurite. The reason for this increase is unclear, however, there is precedent, as the bacterium strain EG13 also has increased biomass production (4.5 fold) in the presence of other metalloid oxyanions (NaVO_3_) compared to metal free medium [45]. It has been suggested that reduction of metal(loid) oxyanions can help dispose of excess electrons through the reoxidation of NADH, FADH_2_, or quinones, therefore retaining optimal redox poise *in vivo* [16,45,46]. This may result in optimal conditions for growth being maintained longer than in the absence of the oxyanion. Possibly, something similar is happening in KR99 and E5. An increase/decrease in biomass resulted in corresponding increased/decreased ATP, as one would expect.

Interestingly, strains E1, E5, KR99, RB3, and RB 16-17 appear to possess similar strategies for tellurite reduction, in both expression and location. While this may imply they are alike, the ability of each strain to reduce and resist tellurite is different [2], which suggests they may actually have different sets of physiological reactions to interact with the oxyanion. Also, one can see reduction profiles in strains RB3 and RB 16-17 differ from the others, with whole cells having a higher rate than fractions even though activity is present in membranes. It is possible more than one mechanism is employed for reducing tellurite and/or some other cellular component is required for maximal effectiveness. Further research is needed to elucidate the exact approach utilized here. Strain E4(1) was the only one requiring a fully functional intact cell, as well as *de novo* protein preparations, for reduction. Other studied species capable of tellurite reduction/resistance at very high levels have been reported to possess a similar requirement [35], and may share a comparable physiology. Possibly, E4(1) has an operational membrane electron transport system similar to *Shewanella oneidensis* MR-1 developed for Fe(III), Mn(IV), and V(V) reduction [47].

In this work, we have established that among different species of AAP, two strategies for tellurite reduction may be required. First, there is a constitutive membrane associated reduction pathway, as seen in E1, E5, KR99, RB3, and RB 16-17. The second, requires *de novo* protein synthesis and fully functional unbroken cells, found in strain E4(1). The identified approaches were not completely unexpected as similarities exist to previously established physiological responses to metal(loid) oxyanions. Membrane associated metal(loid) reduction has been previously observed in *R. capsulatus* and *Enterobacter cloacae* EV-SA01 [16,48]. There is also precedent for the need of fully intact cells [35,49]. It is likely that there are several integral components associated with the outer membrane, periplasm, and inner membrane which are all involved in reducing tellurite. Hence, removal of one component results in loss of function of the entire system. This complex coordinated arrangement is published for reduction of other metal oxyanions [49].

## 5. Conclusions

In summary, this paper broadens what we know about the strategies used by AAP for tellurite reduction and shows that more than one approach has evolved in this physiological group of bacteria to deal with this toxic compound. These strategies differ from the only known example of a tellurite specific reductase system, in Gram positive bacterium *Bacillus* STG-83 [22]. Our investigation has provided a stepping stone for future investigation of the key enzymes and pathways that provide the AAP capability to resist extremely high concentrations of toxic oxyanions.
